# Radiomics signature based on robust features derived from diffusion data for differentiation between benign and malignant solitary pulmonary lesions

**DOI:** 10.1186/s40644-024-00660-4

**Published:** 2024-01-22

**Authors:** Jiaxuan Zhou, Yu Wen, Ruolin Ding, Jieqiong Liu, Hanzhen Fang, Xinchun Li, Kangyan Zhao, Qi Wan

**Affiliations:** 1 Department of Radiology, The Key Laboratory of Advanced Interdisciplinary Studies Center, The First Affiliated Hospital of Guangzhou Medical University, Guangzhou, 510120 Guangdong China; 2 The Second Clinical Medicine School, Guangzhou Medical University, Guangzhou, China; 3 Department of Radiology, Huilai County People’s Hospital, Jieyang, China; 4 Department of Radiology, The Affiliated Hospital of Hubei University of Arts and Science, Xiangyang Central Hospital, Xiangyang, 441021 Hubei China

**Keywords:** Magnetic resonance imaging, Diffusion weighted imaging, Radiomics, Reproducibility, Solitary pulmonary lesion

## Abstract

**Background:**

Classifying and characterizing pulmonary lesions are critical for clinical decision-making process to identify optimal therapeutic strategies. The purpose of this study was to develop and validate a radiomics nomogram for distinguishing between benign and malignant pulmonary lesions based on robust features derived from diffusion images.

**Material and methods:**

The study was conducted in two phases. In the first phase, we prospectively collected 30 patients with pulmonary nodule/mass who underwent twice EPI-DWI scans. The robustness of features between the two scans was evaluated using the concordance correlation coefficient (CCC) and dynamic range (DR). In the second phase, 139 patients who underwent pulmonary DWI were randomly divided into training and test sets in a 7:3 ratio. Maximum relevance minimum redundancy, least absolute shrinkage and selection operator, and logistic regression were used for feature selection and construction of radiomics signatures. Nomograms were established incorporating clinical features, radiomics signatures, and ADC_(0, 800)_. The diagnostic efficiency of different models was evaluated using the area under the curve (AUC) and decision curve analysis.

**Results:**

Among the features extracted from DWI and ADC images, 42.7% and 37.4% were stable (both CCC and DR ≥ 0.85). The AUCs for distinguishing pulmonary lesions in the test set for clinical model, ADC, ADC radiomics signatures, and DWI radiomics signatures were 0.694, 0.802, 0.885, and 0.767, respectively. The nomogram exhibited the best differentiation performance (AUC = 0.923). The decision curve showed that the nomogram consistently outperformed ADC value and clinical model in lesion differentiation.

**Conclusion:**

Our study demonstrates the robustness of radiomics features derived from lung DWI. The ADC radiomics nomogram shows superior clinical net benefits compared to conventional clinical models or ADC values alone in distinguishing solitary pulmonary lesions, offering a promising tool for noninvasive, precision diagnosis in lung cancer.

**Supplementary Information:**

The online version contains supplementary material available at 10.1186/s40644-024-00660-4.

## Introduction

Classifying and characterizing pulmonary lesions are critical steps in the clinical decision-making process to identify optimal therapeutic strategies. The objective of diagnosing and managing pulmonary nodules is to promptly facilitate surgical intervention for all operable malignant lesions, while simultaneously avoiding unnecessary invasive treatment for benign ones. Therefore, accurately distinguishing between benign and malignant nodules in the least invasive manner possible is of paramount importance.

Pulmonary magnetic resonance imaging (MRI) holds significant clinical potential for assessing pulmonary nodules, due to its lack of ionizing radiation and its ability to provide both morphological and functional information [1]. Diffusion weighted imaging (DWI) facilitates the qualitative assessment of tumor cellularity through changes in signal, while also offering quantitative analysis at a cellular level by measuring the apparent diffusion coefficient (ADC). In the context of differentiating between benign and malignant lesions, the conventional single-exponential model ADC has been shown to be comparable to advanced diffusion models, such as intravoxel incoherent motion and diffusion kurtosis imaging [2], and has a similar diagnostic value to PET/CT [3]. Therefore, DWI and ADC show promise as potential biomarkers for the evaluation of pulmonary tumors.

Radiomics has gained traction in recent years for its ability to quantitatively describe tumor phenotypes through the extraction of numerous features from medical images. Its applications have expanded to include lung nodule diagnosis [4] and treatment response evaluation [5]. DWI/ADC imaging, in particular, provides a more nuanced understanding of lesion biological characteristics compared to CT or conventional MR sequences. Previous studies have shown that DWI-based radiomics effectively differentiates between lesion types in regions like the breast [6] and salivary glands [7]. Despite these advancements, there is a current gap in the literature concerning the efficacy of DWI and ADC-based radiomics in distinguishing benign from malignant pulmonary lesions. Specifically, it remains to be determined whether a radiomics model outperforms traditional ADC values alone or if a combination of both offers enhanced diagnostic accuracy.

To address this gap, our study employed DWI and ADC radiomics to differentiate between benign and malignant pulmonary lesions. We began by identifying stable DWI radiomics features through test–retest scanning. This was followed by feature selection and subsequent modeling analysis. Ultimately, we developed a predictive nomogram that integrates radiomics signatures with clinical variables and conventional ADC parameters, aiming to improve the diagnostic differentiation of benign and malignant pulmonary lesions.

## Materials and methods

### Patients

**Fig. 1:**
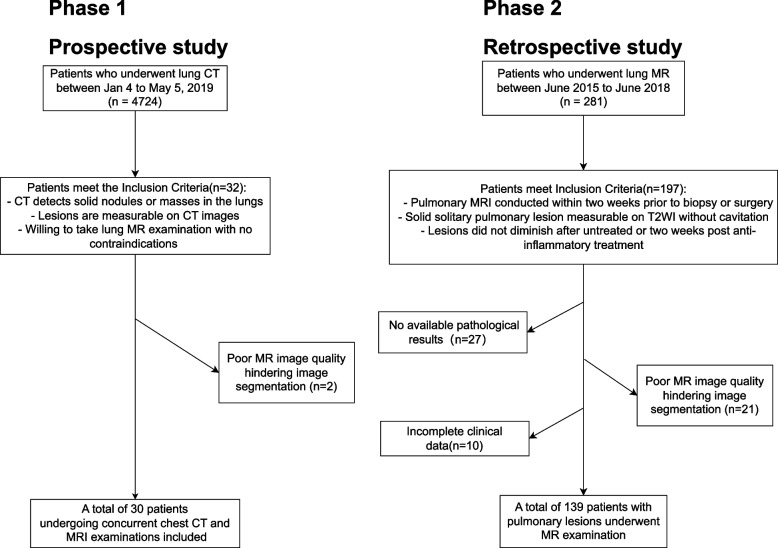
Flow chart for patient recruitment and selection

This study was performed in line with the principles of the Declaration of Helsinki. Approval was granted by the Ethics Committee of the First Affiliated Hospital of Guangzhou Medical University (2018–19). To illustrate the patient inclusion process, a flow chart depicting the stages of patient recruitment and selection is provided in Fig. 1. A checklist for artificial intelligence in medical imaging (CLAIM) [8] were provided in the Supplementary file 1.

In the first phase, we prospectively gathered data from 30 patients undergoing concurrent chest CT and MRI examinations at our institution from January 4 to May 5, 2019. Informed written consent was obtained from all patients who were prospectively enrolled. The inclusion criteria were: (1) the presence of solid nodules or masses in the lungs detected by chest CT, with lesions size measurable on CT images; (2) no contraindications for MR examination. The exclusion criteria was poor MR image quality which hindered image segmentation.

In the second phase, we retrospectively analyzed 139 patients with pulmonary lesions who underwent MR examination at our hospital from June 2015 to June 2018. The following inclusion criteria were applied: (a) pulmonary MRI conducted within two weeks prior to biopsy or surgery; (b) solid solitary pulmonary lesion measurable on T2WI without cavitation; (c) The lesions remained unchanged after remaining untreated or two weeks following anti-inflammatory treatment. The exclusion criteria were: (a) no available pathological results; (b) poor image quality preventing image segmentation; (c) incomplete clinical data. A total of 139 patients were included in the study. There were 97 cases of malignant lesions and 42 cases of benign lesions. The pathology results for malignant lesions included 74 cases of lung adenocarcinoma and 23 cases of lung squamous cell carcinoma. For benign lesions, the results were 13 cases of pulmonary tuberculosis, 7 of infectious granuloma, 7 of organizing pneumonia, 4 of hamartoma, 4 of pulmonary aspergillosis, 3 of pulmonary cryptococcosis, 2 of sclerosing pneumocytoma, 1 of pulmonary sequestration, and 1 of pulmonary glandular fibroma.

### Image data collection

All patients underwent 3.0 T MRI (Achieva, Philips Healthcare, Best, The Netherlands) examination using a body coil array. DWI was performed with a single-shot EPI sequence under free-breathing conditions. In the first phase, DWI was repeated twice, with approximately a 5-min interval, with a repositioning scan conducted prior to the second scan. The b-value was set within the range of 0 to 800 s/mm^2^ with five specific b-values (0, 20, 50, 200, and 800 s/mm^2^). The parameters were as follows: repetition time (TR) = 1195 ms, echo time (TE) = 54 ms, field of view (FOV) 375 mm × 305 mm, slice thickness 5 mm, acquisition voxel size 3 mm × 3 mm × 5 mm, average signal number (NSA) 3, scan time 1 min 5 s. The second phase was a retrospective study, EPI-DWI scanning parameters were TR/TE = 1111 ms/55 ms, NSA 4, FOV 300 mm × 375 mm, matrix 256 × 256, slice thickness/interval = 3.0 mm/0.3 mm, b-value = 0, 5, 10, 15, 20, 25, 50, 80, 150, 300, 500, 800, 1000 s/mm^2^.

### Post-processing of quantitative DWI data

The original EPI-DWI images were transferred to a Philips workstation (Extended MR Workspace 2.6.3.5). The ADC images (ADC_(0, 800)_) were generated by selecting b-values of 0 and 800 s/mm^2^, which were analyzed by two radiologists with 3 and 8 years of thoracic imaging experience respectively. On the ADC images, the solid part of the lesion at its largest plane was selected as the region of interest (ROI), while avoiding areas of liquefactive necrosis, to measure the ADC value. The DWI (b = 800 s/mm^2^) and corresponding ADC_(0, 800)_ maps were exported in DICOM format for image segmentation.

### Lesion segmentation

**Fig. 2:**
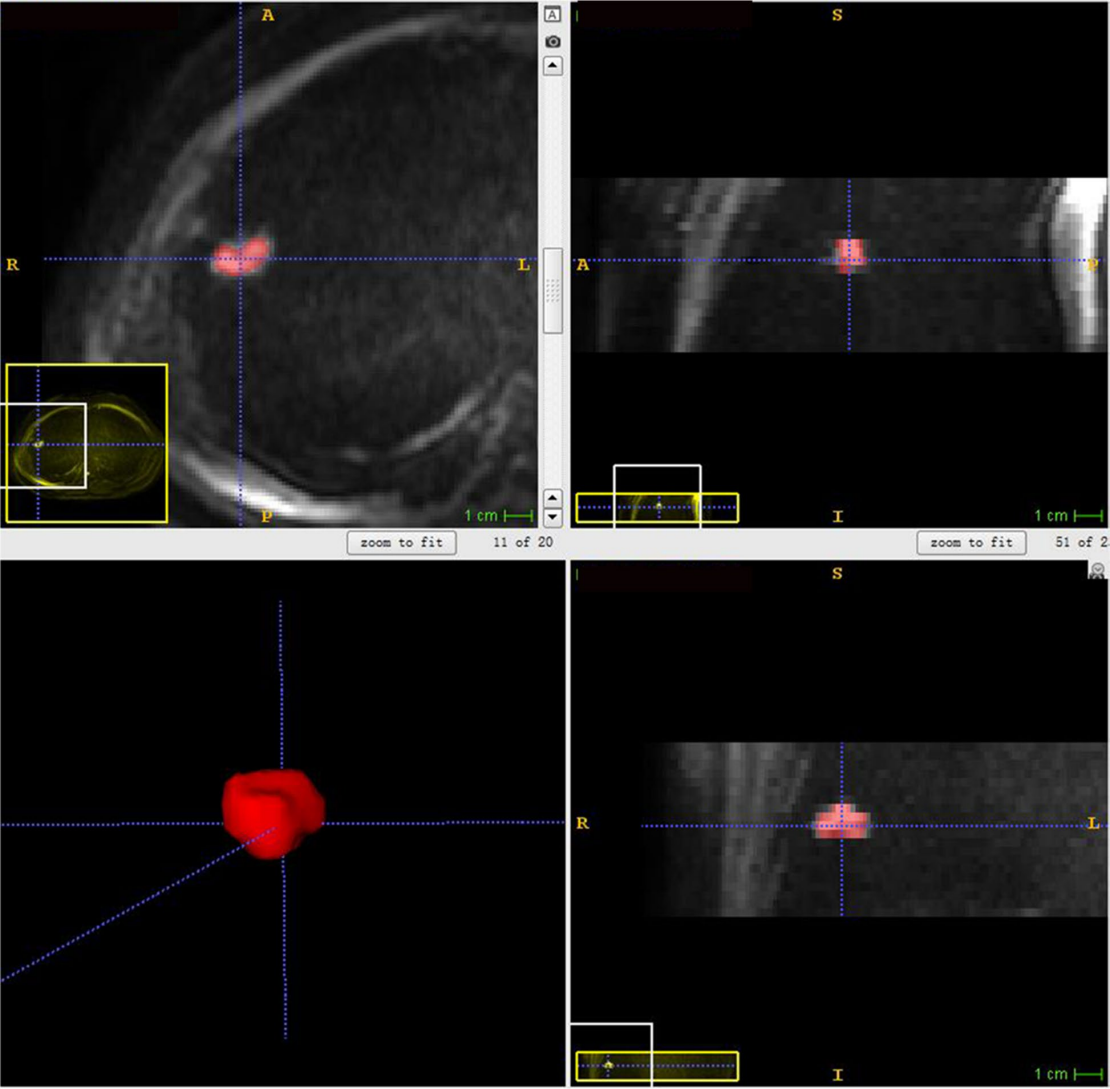
Illustration of the segmentation for a pulmonary nodule located in the upper right lobe on DWI

Lesion segmentation was performed separately on DWI and ADC maps. The open-source software ITK-SNAP (v.3.6.0, http://www.itksnap.org) was used for tumor segmentation (Fig. 2). The ROI covered the entire tumor, excluding visible cavities. Each radiologist independently segmented images from the first scan to assess inter-observer reproducibility. One radiologist segmented the images from the second scan immediately to assess feature stability between scans, while the other radiologist repeated the segmentation on the images from the first scan after 2 months to assess intra-observer consistency.

### ADC value and clinical diagnostic model

A univariate logistic regression model was built to predict the performance of ADC. Multivariate logistic regression was used on clinical factors (including age, sex, site of lesion, lesion size, and smoking history), applying an automated backward elimination method. Stepwise regression iteration identified significant clinical factors to construct the clinical model.

### Radiomics analysis

The DWI_(b=800 s/mm2)_ and ADC_(0, 800)_ maps and their corresponding ROIs were imported into AK software (GE Healthcare), which calculated a total of 396 radiomics features across six categories, including 42 Histogram features, 144 Gy-level co-occurrence matrix features, 10 Haralick features, 180 run-length matrix features, 9 form factor features, and 11 Gy level size zone matrix features.

For the first phase, reproducibility between two scans was assessed using concordance correlation coefficients (CCC) and dynamic range (DR). The intraclass correlation coefficients (ICCs) were used to assess intraobserver and interobserver reproducibility. DR and CCC calculations followed the reference [9]. A DR value close to 1 implies that the feature has a wide biological range and good reproducibility. Increasing differences between repeated scans will lead to a lower DR value. The CCC ranges from 1 to -1, with a value closer to 1 indicating better consistency between the two repeated tests. Robust features for repeated scans were defined as CCC and DR both ≥ 0.85 [10, 11].

For the second phase, the patient dataset was randomly divided into training and test sets at a 7:3 ratio. Z-score was used for feature normalization. The synthetic minority over-sampling technique was used for sample balance in the training set. Stable features (Intra-observer & Inter-observer ICC ≥ 0.75 and DR & CCC ≥ 0.85) were selected. The z-score was then used to normalize the stable features. The maximum relevance minimum redundancy (MRMR) method was used to rank the stable radiomics features. This process allowed us to identify features that were highly relevant to our target outcome while ensuring minimal overlap in the information they provided. The top 30 features were selected and incorporated into the Least Absolute Shrinkage and Selection Operator (Lasso) regression analysis. LASSO was instrumental in refining our feature selection by penalizing less important features, thereby reducing the risk of overfitting and enhancing model robustness. Optimal hyperparameters lambda values were selected for the Lasso regression model via tenfold cross-validation, after which features with P ≥ 0.05 were removed using multivariate logistic regression. The selected radiomics features were input into a multivariate logistic regression analysis, and the regression coefficients of the significant features were weighted to construct the Radscore based on DWI, ADC, and the combined radiomics models.

### Statistical analysis

Quantitative data were expressed as mean ± standard deviation (x ± s). Group comparisons were made using the Mann–Whitney U test for continuous variables and the chi-square test for categorical variables. To account for multiple comparisons, we applied the Bonferroni correction. The significance level was adjusted to 0.005 (0.05/10). The discriminatory ability of each model was assessed by the area under the receiver operating characteristic curve (AUC). Different model comparisons used the DeLong test. The clinical utility of each model was assessed using decision curve analysis (DCA). Radiomics nomograms were constructed based on multivariate analysis results. Statistical analyses were performed using R statistical software (version 3.4.0; R Foundation for Statistical Computing, Vienna, Austria) and Rstudio (version 1.2.1335; RStudio, Boston, MA).

## Results

### Patients characteristics

Subjects with malignant lesions were older than subjects with benign lesions (*p* < 0.005). However, no statistically significant differences were observed in factors such as gender, lesion diameter, location, and smoking history (all *p* > 0.05). There was a similar data distribution between the training and test groups (Table 1).

**Table 1:** Clinical characteristics of patients in the training and test groups

	**Training**			**Test**		
	**Benign**	**Malignant**	*P***-value**	**Benign**	**Malignant**	*P***-value**
**N**	26	72		16	25	
**Age**	48.6 ± 13.6	56.8 ± 10.6	0.002	48.6 ± 13.7	60.4 ± 10.9	0.004
**Diameter(cm)**	3.6 ± 2.5	3.9 ± 1.9	0.579	3.6 ± 2.6	4.8 ± 2.9	0.197
**Gender**			0.368			0.901
** Male**	14 (53.8%)	46 (63.9%)		8 (50.0%)	12 (48.0%)	
** Female**	12 (46.2%)	26 (36.1%)		8 (50.0%)	13 (52.0%)	
**Location**			0.684			0.236
** Upper lobes**	10 (38.5%)	31 (43.1%)		3 (18.8%)	9 (36.0%)	
** Other lobes**	16 (61.5%)	41 (56.9%)		13 (81.2%)	16 (64.0%)	
**Smoke**			0.733			0.354
** Nonsmokers**	16 (61.5%)	47 (65.3%)		10 (62.5%)	19 (76.0%)	
** Smokers**	10 (38.5%)	25 (34.7%)		6 (37.5%)	6 (24.0%)	

### Repeatability and reproducibility of radiomics features

**Fig. 3:**
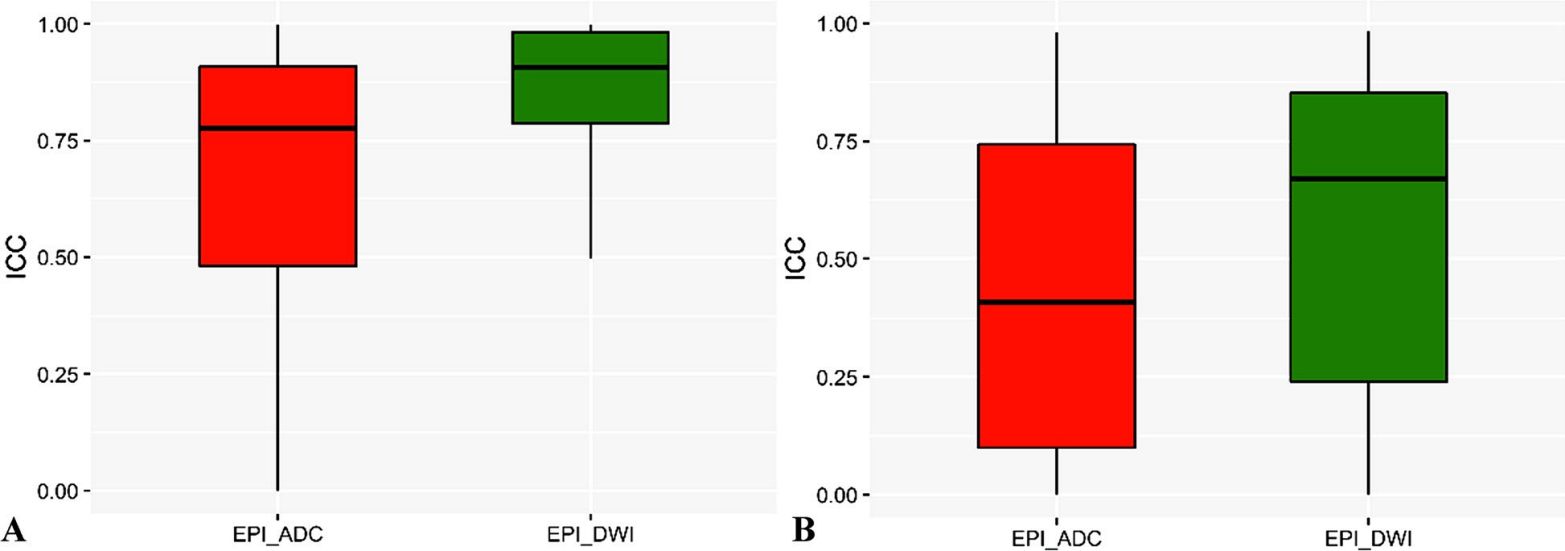
Box and whisker plot depicting the intraobserver **A** and interobserver **B** reproducibility of radiomic features extracted from DWI and ADC images. The plot shows the distribution of intraclass correlation coefficients (ICCs), including the upper extreme, upper quartile, median, lower quartile, and lower extreme

In repeated scanning, the proportion of repeatable features was slightly higher for DWI (*n* = 169/396, 42.7%) than for ADC (*n* = 148/396, 37.4%) (Table 2). Intra-observer reproducibility was superior to inter-observer reproducibility, and the ICC of DWI radiomics features was superior to that of ADC (Fig. 3).

**Table 2:** Robust features of different sequences and their proportions in corresponding feature classes

	CCC&DR	Formfactor (*n* = 9)	GLCM (*n* = 144)	GLSZM (*n* = 11)	Haralick (*n* = 10)	RLM (*n* = 180)	Histogram (*n* = 42)	Total (*n* = 396)
EPI ADC	≥ 0.85	5 (55.5%)	32 (22.2%)	3 (27.2%)	2 (20%)	90 (50%)	8 (19.0%)	148 (37.4%)
	≥ 0.90	5 (55.5%)	19 (13.1%)	3 (27.2%)	2 (20%)	59 (32.7%)	5 (11.9%)	93 (23.5%)
	≥ 0.95	5 (55.5%)	3 (2.0%)	2 (18.1%)	0 (0%)	33 (18.3%)	5 (11.9%)	47 (11.9%)
EPI DWI	≥ 0.85	9 (100%)	58 (40.2%)	3 (27.2%)	8 (80%)	83 (46.1%)	16 (38.0%)	169 (42.7%)
	≥ 0.90	9 (100%)	50 (34.7%)	3 (27.2%)	7 (70%)	36 (20.0%)	5 (11.9%)	110 (27.8%)
	≥ 0.95	5 (55.5%)	27 (18.7%)	2 (18.1%)	4 (40%)	31 (17.2%)	4 (9.5%)	74 (18.7%)

### Diagnostic performance of multiple models

After multivariate logistic regression screening, the clinical model included age as an analytical factor. The area under the curve in the training and test groups were 0.696 (95% CI, 0.575–0.818) and 0.694 (0.524–0.864) respectively. The ADC_(0, 800)_ for malignant tumors was 1.154 ± 0.286 × 10–3 mm^2^/s, and it was 1.576 ± 0.409 × 10–3 mm^2^/s for benign lesions. The difference was statistically significant (*P* < 0.001). The AUC of ADC in the training group was 0.785 (0.680–0.890), and in the test group, it was 0.802 (0.666–0.937).

The ADC radiomics model included a total of 6 features, with an AUC of 0.902 (0.860–0.943) in the training group and 0.885 (0.774–0.997) in the test group. The DWI radiomics model included a total of 5 features, with an AUC of 0.850 (0.796–0.904) in the training group and 0.767 (0.578–0.957) in the test group. The combined ADC + DWI radiomics model included 7 features, with an AUC of 0.812 (0.752–0.871) in the training group and 0.670 (0.467–0.873) in the test group (Table 3). The feature selection and model construction for ADC, DWI, and combined radiomics analysis were detailed in the Supplementary file 2.

**Table 3:** Diagnostic performance of different models

	**Train-AUC**	**Test-AUC**
Radiomics(ADC)	0.901(0.860–0.943)	0.885(0.774–0.996)
Radiomics(DWI)	0.850(0.796–0.903)	0.767(0.578–0.956)
Radiomics(ADC + DWI)	0.812(0.752–0.871)	0.670(0.467–0.873)
Clinical model	0.696(0.575–0.818)	0.694(0.524–0.864)
ADC	0.785(0.680–0.890)	0.802(0.666–0.937)
Nomogram	0.858(0.780–0.936)	0.923(0.842–1)

95% confidence interval are in parentheses

**Fig. 4:**
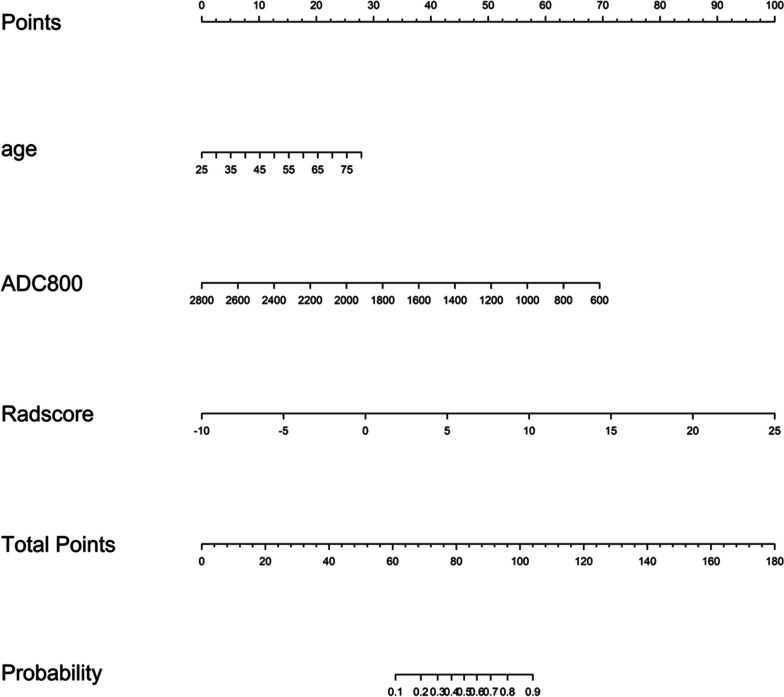
A constructed radiomics nomogram incorporating three variables: patient age, ADC value at b = 800 s/mm2 (ADC800), and radiomics score (Radscore). The 'Total Points' represent each patient's score computed based on the three variables, while 'Probability' signifies the malignant probability of the lesion

**Fig. 5:**
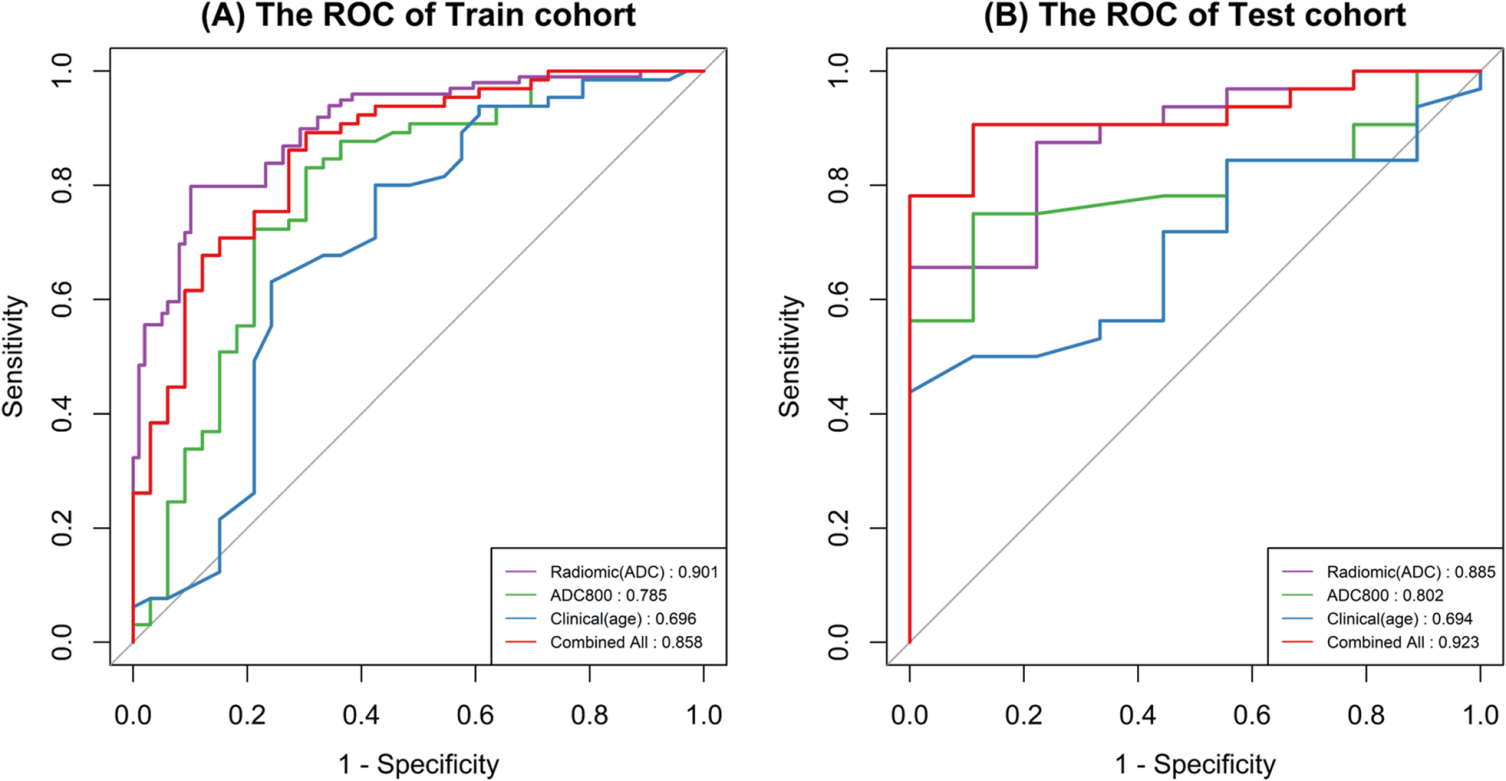
Comparative analysis of the diagnostic efficacy between the developed nomogram (combined all) and clinical, ADC800, and ADC radiomics models in both the training (**A**) and test (**B**) groups

The results of the multivariate logistic regression analysis showed that in the training samples, age, ADC800, and Radscore (ADC) were independent risk factors for predicting the benignity or malignancy of lung lesions. A radiomics nomogram was constructed based on the results of the multivariate analysis (Fig. 4). The diagnostic performance of multiple models was shown in Fig. 5.

**Fig. 6:**
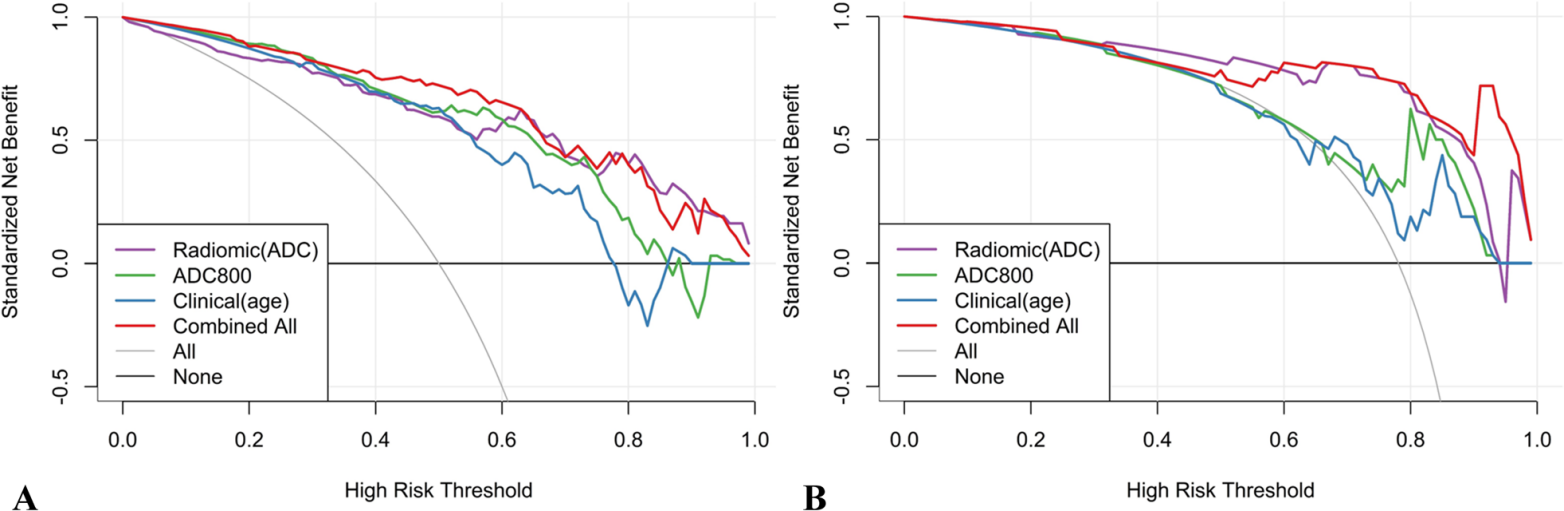
Decision curve analysis for the developed nomogram (combined all), clinical, ADC_(0, 800)_ value, and ADC radiomics model in both the training **A** and test **B** groups

The Delong test showed that there was no statistically significant difference in performance between the ADC radiomics model, the DWI radiomics model, and the combined model (*p* > 0.05 for all) (Table 4). The decision curve showed the combined model (nomogram) consistently show a higher net benefit compared to ADC and clinical models. (Fig. 6).

**Table 4:** Delong test results of different models

	**Radiomic (ADC)**	**Radiomic(DWI)**	**Radiomic (ADC + DWI)**	**Clinical model**	**ADC**	**Nomogram**
Radiomic(ADC)	1	0.36	0.060	0.076	0.343	0.449
Radiomic(DWI)	0.360	1	0.477	0.556	0.741	0.160
Radiomic(ADC + DWI)	0.060	0.477	1	0.864	0.253	0.016
Clinical model	0.076	0.556	0.864	1	0.377	0.007
ADC800	0.343	0.741	0.253	0.377	1	0.060
Nomogram	0.449	0.160	**0.016**	**0.007**	0.060	1

## Discussion

To the best of our knowledge, this is the first study to develop and validate a radiomics model for differentiating benign and malignant pulmonary lesions based on the robust test–retest features in DWI and ADC images. Our findings indicate that radiomics signature can effectively differentiate between benign and malignant pulmonary lesions, and a nomogram that combines radiomics, clinical information, and ADC values offers a significantly better clinical net benefit, outperforming both ADC and clinical models alone.

Clinical features including age, gender, lesion location of the upper lobe, and smoking history were collected due to their documented relevance in lung cancer occurrence [9, 10]. Our findings indicated that patients with malignant lesions were significantly older than those with benign lesions, yet no significant differences were observed for gender, smoking history, or lesion size. This may be indicative of the growing prevalence of lung cancer among non-smoking women, potentially influenced by factors such as environmental tobacco smoke, cooking fumes [11], and air pollution exposure [12, 13]. Of note, the benign group in our study predominantly consisted of mass-like inflammatory lesions, which are clinically challenging to distinguish from malignant lesions. Consequently, the utility of a diagnostic model based solely on clinical features or lesion size may have limitations.

ADC values, serving as a crucial parameter for differentiating benign and malignant pulmonary lesions, were calculated in this study using b = 0 and 800 s/mm^2^, as suggested by previous research [2]. Notably, both training and test groups revealed significant ADC differences between benign and malignant lesions. Typically, the malignant display lower ADC values due to dense cellular structures limiting water molecular diffusion, whereas benign lesions are more likely to demonstrate higher ADC [14].

Previous studies have demonstrated that breathing states significantly impact the reproducibility of CT image features acquired through repeated scans of lung cancer patients [15]. In this study, DWI was acquired during free breathing, a methodology commonly employed in most current lung DWI studies; The proportion of stable features in DWI was slightly higher than in ADC, which is consistent with a previous phantom study [16]. Our results showed 42.7% of EPI-DWI features remained stable (CCC and DR ≥ 0.85) upon repeated scanning, with features presenting CCC and DR ≥ 0.9 accounting for 27.8% of the total. This is comparable to a previous study assessing the reproducibility of lung CT radiomic features using scan-rescan images, where 30.14% of the features were considered stable (CCC ≥ 0.9) [17]. This indicates the feasibility of conducting a radiomics analysis based on free-breathing lung DWI.

This study established stable features in a prospective cohort and validated the value of these stable features for pulmonary lesion differentiation in another independent cohort. Surprisingly, despite the disparity of scanning parameters between the two cohorts, the radiomics model based on stable features still demonstrated excellent performance. This robustness in performance could suggest the stability of the ADC radiomics, consisting with a previous study demonstrating the stability of ADC-based radiomics features across multiple centers, field strengths (1.5 T—3 T) and MRI vendors [18]. However, a recent study on bone marrow using T1WI and T2WI in a multi-MRI-scanner test–retest scenario indicated that only a limited number of radiomics features are reproducible with different MRI sequences or scanners [19]; this might raise concerns for the generalizability of single-scanner studies; however, it's important to note that the sequences and imaging locations in this study [19] differ from those in our research. In addition, although some prior studies have shown that ADC exhibits good repeatability across different institutions and MRI vendors [20, 21], some other studies on bone marrow and prostate [22, 23], have shown significant variability in ADC reproducibility. These contrasting findings highlight the intricacies of ADC analysis and suggest that factors such as different anatomic locations or tissues [24] and the measurement methods (2D or 3D) may contribute significantly to the variability of ADC reproducibility. This underscores the importance of reproducibility studies that incorporate multiple measurement and test–retest methods when conducting ADC or ADC radiomics research across various anatomical sites.

Our study achieved promising results by utilizing radiomics features that exhibited high reproducibility across different scans and observers, with both intra- and inter-observer ICCs of ≥ 0.75 and CCC and DR values of ≥ 0.85. This provided a degree of confidence in the stability and reliability of our results. Notably, the ADC radiomics model outperformed ADC values alone in diagnostic efficiency, also yielding a higher clinical net benefit in most cases. The enhanced performance can be attributed to the comprehensive information extracted from whole-tumor segmentation, which provides a nuanced evaluation of lesions by capturing tumor heterogeneity, cell density, and microenvironmental factors. However, the combined radiomics model, incorporating both ADC and DWI features, did not demonstrate superior performance over models based on individual sequences. This lack of additive benefit may be due to the inherent relationship between ADC maps and DWI; their textural features could contain redundant information that dilutes the efficacy of the combined model.

We employed MRMR followed by LASSO for feature selection, capitalizing on MRMR's ability to identify relevant, non-redundant features and LASSO's strength in refining and regularizing the feature set. This sequential approach aligns with recent radiomics research [25], which effectively balances the need for predictive accuracy and avoiding overfitting in our model development. In the construction of the nomogram, we opted for the ADC radiomics signature, which showed superior performance among the three radiomics signatures, to eliminate redundancy. We included both ADC values and the ADC radscore in the nomogram to provide a more comprehensive assessment. While manually measured ADC values capture localized cellular density, ADC radiomics offer insights into the overall tumor morphology and heterogeneity, thus complementing each other. A breast cancer study [6] also demonstrated that incorporating manually measured ADC values could enhance the predictive capability of a radiomics nomogram for distinguishing between benign and malignant lesions.

Some limitations exist in this study. First, our radiomics model was built upon features that demonstrated stability across different observers and repeated scan sequences, potentially enhancing the model's generalizability. However, it is important to acknowledge that the investigation of feature stability through a single-scanner test–retest approach might primarily ensures model reliability for the specific scanner used. The generalizability of this model in a multicentric context remains to be validated. Therefore, further research involving multicenter studies is necessary to confirm the model's reliability in diverse clinical settings. Second, we only included DWI at b = 800 s/mm^2^ and ADC_(0, 800)_ in our analysis, excluding the Intravoxel incoherent motion (IVIM)-derived parameter maps. This is because ADC values are more accessible and clinically relevant. Future research could consider incorporating IVIM parameters into radiomics analyses. Thirdly, while our model demonstrated efficacy in larger pulmonary tumors, its applicability to smaller pulmonary nodules, particularly those under 1 cm as outlined in the Fleischner Guidelines [10], might be compromised.

## Conclusion

In summary, our study demonstrated the robustness of lung DWI radiomics features which signals a promising direction for clinical implementation of ADC-based radiomics in pulmonary lesion assessment. The ADC radiomics nomogram has superior clinical net benefits compared to conventional clinical models or ADC values alone in distinguishing solitary pulmonary lesions, offering a promising tool for noninvasive and precision diagnosis of lung cancer.

### Supplementary Information

**Additional file 1.**

**Additional file 2.**

## Data Availability

The datasets used and/or analyzed during the current study are available from the corresponding author on reasonable request.

## Abbreviations

AUC: Area under the curve
ADC: Apparent diffusion coefficient
CCC: Concordance correlation coefficients
DR: Dynamic range
DWI: Diffusion-weighted imaging
EPI: Echo-planar imaging
IVIM: Intravoxel incoherent motion
ICC: Intra-class correlation coefficient
DCA: Decision curve analysis
