# Supplementary material for: Radiomics signature based on robust features derived from diffusion data for differentiation between benign and malignant solitary pulmonary lesions
Source: Cancer Imaging. 2024 Jan 22;24:14. doi: 10.1186/s40644-024-00660-4 (PMC10802010; doi:10.1186/s40644-024-00660-4)
Supplement: Supplementary file 2 — Additional file 2. [file 40644_2024_660_MOESM2_ESM.docx]

**Radiomics feature selection and model construction for ADC, DWI, and combined radiomics analysis**

Through the use of MRMR (Maximum Relevance Minimum Redundancy) algorithm, LASSO (Least Absolute Shrinkage and Selection Operator), and multivariate logistic regression for dimension reduction and feature selection, radiomics models based on ADC, DWI, and their combination were constructed.

For the ADC radiomics model feature selection, initially, 69 stable ADC radiomics features were selected based on the results of the phase 1 stability study. The MRMR algorithm then selected the top 30 features for LASSO (tenfold cross-validation), leaving 14 features (Figure S1). Subsequent multivariate logistic regression removed features with P ≥ 0.05, leaving 6 features. The final ADC radiomics model incorporated these 6 features. The feature names and calculation formulas are as follows:

Radscore (ADC) =0.125+10.838*SizeZonevariability–6.728* RunLengthNonuniformity_angle135_offset1-4.035*GLCMEntropy_angle90 _offset7+4.653*GLCMEntropy_angle0_offset7–3.651*Maximum3DDiameter-1.417*Quantile0.75。


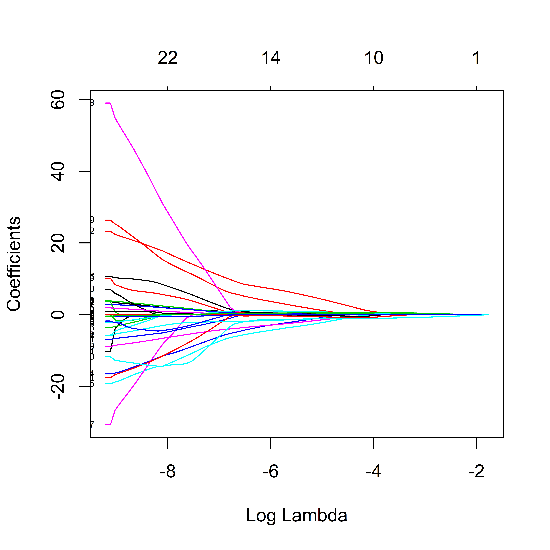

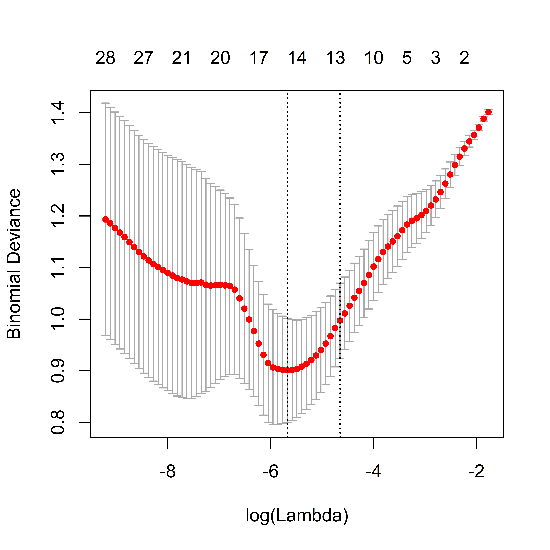


Figure S1. Feature selection and dimension reduction via least absolute shrinkage and selection operator(lasso)algorithm for ADC radiomics model. The left figure shows the coefficient profiles of 30 radiomics features against the log(lambda). The right figure is the cross-validation curve. The 10-fold cross validation method is used to select the adjustment parameters(lambda)in the lasso model.

For the DWI radiomics model feature selection, initially, 89 stable DWI radiomics features were selected based on the results of the phase 1 stability study. The MRMR algorithm then selected the top 30 features for LASSO (tenfold cross-validation), leaving 25 features (Figure S2). Subsequent multivariate logistic regression removed features with P ≥ 0.05, leaving 6 features. The final DWI radiomics model incorporated these 6 features. The features and calculation formulas are as follows:

Radscore(DWI)=0.326+15.767*Range–15.145MaxIntensity–1.844* InverseDifferenceMoment_angle45_offset1–1.037*HaralickCorrelation_AllDirection _offset7_SD+0.536*LongRunEmphasis_angle45_offset7+0.221*GLCMEntropy_angle45_offset7


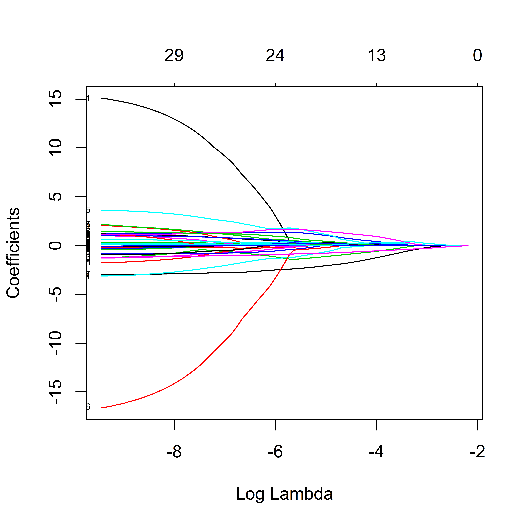

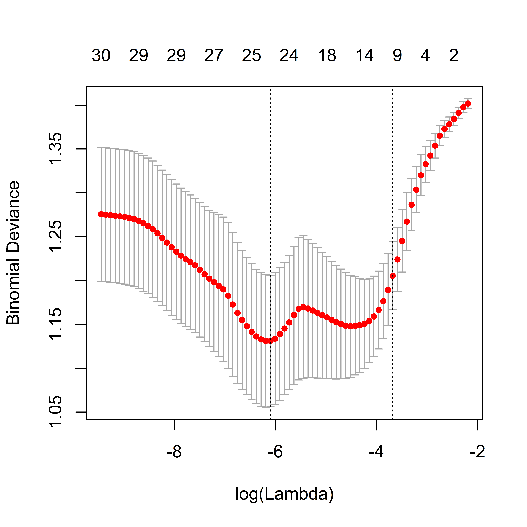


Figure S2. Feature selection and dimension reduction via least absolute shrinkage and selection operator (lasso) algorithm for DWI radiomics model. The left figure shows the coefficient profiles of 30 radiomics features against the log (ambda). The right figure is the cross-validation curve. The 10-fold cross validation method is used to select the adjustment parameters (lambda) in the lasso model.

For the combined ADC+DWI radiomics model feature selection, initially, 158 stable radiomics features were selected based on the results of the phase 1 stability study. The MRMR algorithm then selected the top 30 features for LASSO (tenfold cross-validation), leaving 14 features (Figure S3). Subsequent multivariate logistic regression removed features with P ≥ 0.05, leaving 7 features. The final combined ADC and DWI radiomics model incorporated 5 features. The features and calculation formulas are as follows:

Radscore (ADC+DWI) = –0.00985+0.8264*DWI_Inertia_angle90_offset4 – 1.0827* ADC_HaraEntroy + 0.8799*D_LongRunEmphasis_AllDirection_offset4 +0.7432*ADC_InverseDifferenceMoment_angle0_offset1+0.8660*ADC_GLCMEntropy_angle90_offset4–1.0199DWI_LongRunHighGreyLevelEmphasis_AllDirection _offset1-0.7129*DWI_HaralickCorrelation_AllDirection_offset7_SD


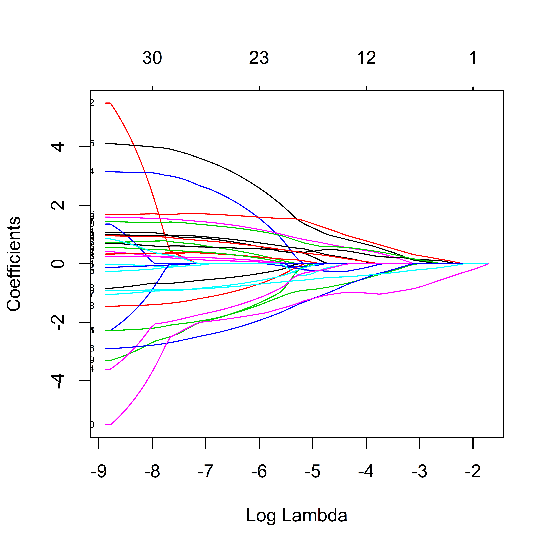

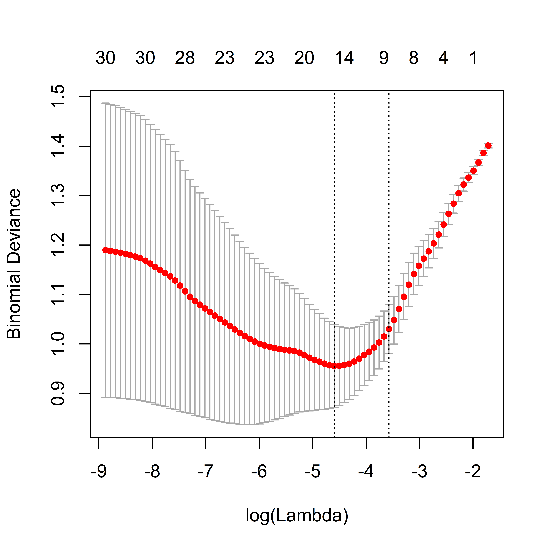


Figure S3. Feature selection and dimension reduction via least absolute shrinkage and selection operator (lasso) algorithm for combined ADC and DWI radiomics model. The left figure shows the coefficient profiles of 30 radiomics features against the log (lambda). The right figure is the cross-validation curve. The 10-fold cross validation method is used to select the adjustment parameters (lambda) in the lasso model.
